# Lessons for a digital future from the school of the pandemic: From distance learning to virtual reality

**DOI:** 10.3389/fsoc.2022.1101124

**Published:** 2022-12-16

**Authors:** Maria Paola Faggiano, Antonio Fasanella

**Affiliations:** Department of Communication and Social Research, Sapienza University of Rome, Rome, Italy

**Keywords:** distance learning, pandemic, secondary school, educational inequalities, digital technologies, VR technology, digital research, web survey

## Abstract

The unexpected onset of the pandemic emergency placed so-called Distance Learning (DL) at the center of the academic world, affecting students and teachers across all formative steps. The DL experience has opened up the way for many queries in terms of research on the front of education, besides showcasing instances of innovation within the schooling institution, both increasingly urgent and no longer deferrable. The collective shock that started in March of 2020 was an opportunity to incentivize a leap in evolution, heavily digital in nature, within the educational system; howbeit, the generation of digital natives were already, prior to the onset of COVID-19, waiting to sense greater openness in the Italian school system toward newer technologies, in addition to less standardized, more innovative, creative and hybrid didactic formulas. In the presented study–a web survey launched in the spring of 2021–a large sample of students were invited to retrace their experience with DL, and express their relating assessments and reviews. Conducting the entirety of the study remotely turned out to be a winning data collection technique given a situation, comparable to the one experienced globally, in which face-to-face meetings had become impossible. Through in-depth analysis of the different contexts–social, cultural, technological, spatial, relational–in which the DL experience took hold, this contribution holds the purpose of illustrating the main DL adaptation profiles of the sample reached, valorizing the perceptual dimension, through the systematic comparison of online and in-person didactics. Analysis of the identified forms of adaptation created an opportunity to reconstruct the image of school that the interviewees held, how much they valued it, the trust they placed therein, the developments they predicted and desired for the institution. Focusing–responsibly, and taking stock of the possible ethical implications–on the future opportunities held by technological progress, in itself boosted by the pandemic, are located within a wider experimentation of VR-equipped classrooms, in a multidisciplinary perspective, offering a concrete solution to the needs of both students and teachers.

## Introduction

This contribution highlights a portion of the data within a broader dataset, with the intention of identifying the main *adaptation profiles to Distance Learning (DL)* within a *sample of Italian secondary school students*. Before venturing into the description of the research instrumentation devised, before illustrating the survey findings and drawing the opportune conclusions, it is important to describe the scenario in which the study is located, and the premises from which it stems. Not before, however, having stressed that the reference participants, from their varyingly solid socio-economic and cultural backgrounds, and with differing degrees of support from educational figures in their households, and in their scholastic and extra-scholastic environments, were called to face and adapt to the living conditions dictated by the pandemic, in a very delicate existential phase that is anything but linear, in which the formation of personal and social identity requires a great deal of energy (see Adnan and Anwar, 2020; Aucejo et al., 2020).

In the face of a potential range of the youths' reactions to the ongoing transition in the academic world–and partly due to the participants' differing and preceding cognitive, emotional and social background–presented herein is empirical evidence concerning the differences perceived by the interviewees in in-person and distance learning, taking for granted that, for a large portion of students, and by and large transversally, the advent of *Distance Learning* reduced cognitive-emotional involvement in didactic activities, as well as abruptly, and at times dramatically, depleting opportunities for interaction and relations on different levels (see Almahasees et al., 2021).

Much of the scientific debate around DL has focused on the issue of *worsening social inequalities* and the *accentuation of preexisting gaps* in the school system, and in broader society likewise. Admittedly (and the empirical evidence herein confirms this picture), notwithstanding all the effort in the direction of digitalizing didactics, in a school system busy with generating *emergency training solutions*, there were those who–already struggling (due to social extraction and technological endowment/access to technology) –were left even further behind and experienced particularly critical forms of hardship (Ciurnelli and Izzo, 2020; Lombardo and Mauceri, 2020; Nuzzaci et al., 2020; Ghigi and Piras, 2021; Istituto Toniolo, 2021; Saraceno, 2021).

Whereas, however, it is true that DL, in its expression, caused trouble especially to those who were already struggling, another observation cannot likewise be ignored: *the assessment of the DL experience* (and the consequent form of individual adaptation to it) *on part of adolescents*, notoriously defined as *digital natives, also depends on their starting digital competencies*, presumably already advanced and extensive in the pre-pandemic stage. On the other hand, in the face of *remotization* and radical transformation of broad spheres of social activity–school, free time, social life etc. –in response to a system of restrictions aimed at stopping the spread of the Coronavirus (Mancaniello, 2020), the effects of which have essentially rolled over into the current post-pandemic phase, the interest in the challenges and opportunities, and in the pitfalls concerning the use of digital technology during adolescence, cannot but gain substance and beget new research questions (Riva, 2014, 2016). It is, in fact, evident how technology, already at the center of digital natives' lives, took on a pervasive character throughout the pandemic, and youngsters spent much of their *daily lives before a screen*, their existence increasingly characterized by ever-extensive usage of electronic devices. In the face of this, the contribution, therefore, has the objective of investigating how, in such a scenario, “remote” student-life took shape. This led to the interest in carrying out an observation empirically founded on the opportunities and risks connected with the use of platforms, in a scenario where the digital burst-out in schools was compounded by a preexisting, deep-seated usage of the digital (the *touch generation–native speakers* of netspeak, and the language of social networks and videogames–widely represented in our survey sample, are notoriously characterized by an ability to interact with technological devices that precedes any approach to reading or writing), especially for entertainment and recreation (consider the use of social platforms, notably Instagram, or *gaming* activities).

Evidently, in the life of the interviewed teenagers, *Information and Communication Technologies (ICT)* play a major role, along with family, school, and other significant collective subjects, rightfully entering the realm of socialization agents in contemporary digital society, in the face of their ample contribution in terms of the reproduction of symbols, representations, outlooks and cultural products of reference. Technology redefines the boundaries of bodies and space, as well as the way emotions are experienced; it multiplies networks and bonds, creates new forms of belonging and unprecedented communal bonds; it reconfigures the sense, structure and distribution of social capital; it hybridizes spaces (real-virtual), spheres (public-private), existence and relations (online-offline). Therefore, it appears essential to dwell, among other aspects, on the media diet of the interviewed adolescents, analyzing the range of technologies and services to which they have access, and partly in reaction to the upheaval on the back of the COVID-19 pandemic, while also paying mind to the nature of the familial context, and the quality and sturdiness of the relations therein. In such a scenario, one may suppose that a large chunk of *youngsters thrown into DL*, used to aptly navigating platforms long before the onset of COVID-19, possessed *digital competencies* suited to reviewing the didactics produced throughout the pandemic. In view of this, the assessment of DL on part of the reached students (see cfr. Fasanella and Faggiano, 2022; Faggiano and Mauceri, 2022), besides calling on well-known dynamics connected with social stratification, and with the effects of a social life stripped of face-to-face interaction, should take into account a cognizant, familial and widespread usage of platforms (especially those for entertainment purposes, or, at any rate, non-scholastic) on part of the young, which predates the pandemic. In this sense, a critical evaluation of DL–presuming an analysis of the students' teacher evaluations filtered, so to speak, through a “high bar”–could also be connected to 1. *the little appeal* held by e-learning platforms, hypothetically defined by digital natives as a tool for issuing didactics that is “far removed” (obsolete, poorly interactive etc.) from their daily lives; 2. the e-learning platform usage by teachers (not by chance referred to in literature as *digital immigrants*–Prensky, 2001), perhaps “clumsy” players in an academic scenario that was now suddenly remote and radically different from the “real life” one wherein, hitherto, they had moved as experts, with the utmost ease and confidence^1^. In this perspective, DL could be compared to a “hastily tailored coat” sewn around the faculty, in many instances not fully up to par in the students' eyes, probably focused–throughout the pandemic–more on the available instruments for the transfer of knowledge/competency (the *means*), on the *usage of the means* and the *packaging of content*, rather than on the content itself (Pitzalis et al., 2016; Giancola et al., 2019).

All the latest studies on *online education*, see the efficient use of digital technologies for educational purposes combined with meticulous planning and preparation groundwork, aimed at engaging students and holding their attention, a collaborative working style of a reciprocal nature, along with the production of quality didactics, potentially capable, even, of exceeding the apprehension results associated to traditional didactics (see GarcíaBotero et al., 2018; Bower, 2019; Gonzalez et al., 2020; Hodges et al., 2020; Nguyen et al., 2021). Nonetheless, although it presented a challenge, DL was launched without the teachers having time to learn, in a reasoned and gradual manner, efficient transformation and adaptation strategies for their teaching style, without modifying the educational objectives, or the expected results^2^. The abrupt interchange, therefore, took place in a way that prevented educators from adequately designing an online education that was able to mitigate the negative effects of the digital transition on the students' *cognitive engagement* and *cognitive absorption*, of which the salient ingredients are *attention, interest/curiosity, concentration* (along with the “springs” needed to activate them (Saade and Bahli, 2005; Kemp et al., 2019). Moreover, the emergency did not give students and teachers the time to identify, tweak and test alternative communicative-relational models, suitable for transforming digital environments into spaces that efficiently express learning models based on participation and cooperation (Weick and Sutcliffe, 2011); the aforementioned was not free of repercussions on the expected learning results (Bower, 2019), on the overall psycho-emotional state of the actors involved–especially with a view to relations between peers, and between students and educators -, on the conventional achievement of educational objectives^3^.

Some observations on the world of *social networks* and *gaming* allows further development of the initial introductory framework in this contribution. The former are a privileged dimension in the daily activity of digital natives, for the purpose of creating hybrid social networks straddling the online and offline dimensions (Riva, 2016). The offer of *networking* platforms is now very extensive, and with that, the instruments the younger generation have to express and represent themselves are multiplying. Images and videos become an optional vessel for sponsoring oneself; meanwhile, the offer of increasingly updated applications grows, configured, nowadays, as the instruments to construct one's personal and social identity: from video editing *tools* to retouching options for pictures, from post-production applications to emojis, and so on. Instagram, namely, is the most widely used and appreciated network when it comes to teenagers sharing visual content, from existential images, to the expression of one's individual and social portent. As known, the archive and functions to share videos and images with followers, on said platform, combine with comments, the upload of temporary stories, tags, hashtags; the latter, as thematic aggregators, are keywords, issues, overarching interests, specific virtual communities. The *gaming* dimension completes the *digital identikit* of the world of the reference youth group. Its main elements of appeal can be so classified: 1. *Interactivity* (dynamic, creative and strategic roles during gameplay; challenges experienced in the first person; personal contribution to the course of the game); 2. *Immersion* (relating to characters/mission of the game; complex storylines; emotional impact); 3. *Simulation* (even when set in a fantastical setting, the game presents such a level of detail that the player, becoming immersed in the portrayed situation, can experience situations that near reality, be it a journey into space, piloting a racecar or the adventures of a superhero); 4. *Shared gaming experience in a hybrid space/Socializing function* (in terms of reenforcing preexisting friendships and/or the possibility of forming new ones, the realization of forms of conversation and interaction carried on by extroverted subjects, and shy, more reserved profiles alike). Several are the benefits and positive aspects connected with the dimension of *gaming*: the existence of complex and enigmatic storylines can run concurrently to the development of logical thinking, incentivize the capacity to devise strategies and identify solutions to problems, it can affect creativity; the presence of *group tasks* reinforces the sense of belonging and the propensity to collaborate with others, exposure to different cultures, be they real or imaginary, and other identities. It is not by chance that the term *gamification* refers to a tendency, that is increasingly gaining traction, to import rules, techniques and methods from the gaming world into other dimensions, for example education, with the purpose of intensifying and improving–as regards the end user–the communicative effectiveness of content, interest and curiosity, enjoyment (why not have fun while learning at school?), *engagement*, learning/assimilation opportunities, interaction dynamics. In this regard, experimenting with digitally-supported innovative didactic methods–of which there is no shortage of concrete examples in both the pre-pandemic and pandemic stages–could represent a stimulus for the future implementation of participatory education models, capable of fostering and reinforcing critical thinking, the desire to delve deeper and the development of basic, transversal competencies on part of the students.

It is abundantly evident that the world of social networks and online games, here referred to as it constitutes a precious reference model for designing the didactics of the future, besides the advantages and opportunities, carries risks and snares. It will suffice to consider situations in which a teenager may invest excessive temporal or cognitive resources, or worse still, fall into veritable forms of *digital addiction* (Mauceri and Di Censi, 2020), and/or *social isolation-withdrawal*. The restrictive measures in place to contain the pandemic, and the consequent social isolation, on the one hand, in combination with DL, with an end to avoiding the interruption of scholastic-training programs, have incontrovertibly contributed to increasing the time spent on digital devices/platforms. The trait of *hyper-connection* is certainly not risk-free (in terms of the reference target, a red flag is constituted by the decline in academic performance), including the rise in aggressive behavior, a wide array of forms of psychological distress, the frantic search for confirmation and virtual admiration, body shaming, cyber-bullying (Quwaider et al., 2019; Li et al., 2020; Rudolf, 2020). Only a trained and cognizant use of platforms can protect these youngsters from snares and from drifting, and from a dysfunctional use of DL, and insufficiently innovative post-pandemic didactic formulas. This brings into question the main socialization agencies around adolescents, parents and teachers first of all, who represent–facts at hand–a generation that is still too distant from their younger counterpart in terms of digital competencies and penchant for innovation. These role models are the ones holding the key to these youngsters' future, provided they prove themselves more willing to “make their own”–in a constructive and reciprocal manner–the daily practices of the young: 1. educators, by way of a more active contribution to the digital switch, not just in terms of expanding their digital competencies, but simultaneously implicating greater openness and empathy toward the young, for whom to develop and systemize the willingness to meet needs, innovative solutions and tools for designing the school of tomorrow (see Wang et al., 2021); 2. parents, again by way of closing the gap in terms of the digital natives generation, with a view to a more careful monitoring, and more active participation in the lives of their children, who are inevitably at risk in an increasingly digitalized world, and who may plausibly switch off their cameras during a boring lesson, preferring an immersion–with no regard for time–in a non-academic virtual experience, considered more appealing, and more important to the self.

Given these premises, our question, also taking into account the possible ethical implications, is whether, among the future opportunities for the world of school–born out of a technological progress, in itself boosted by the pandemic –, a more widespread experimentation of VR-equipped classrooms could appear, thus identifying concrete solutions to the needs of both students and teachers students and teachers, in a multidisciplinary perspective.

## Materials and methods

The study from which the present contribution^4^ stems, carried out nationwide at the height of the pandemic (spring of 2021), is a *closed web survey targeting a specific population* (see Mauceri et al., 2022), funded by Sapienza University in Rome, which comprised 209 of the 1,599^5^ institutions in Italy's region and province capitals (calculated return on percentage on academic institutions: 13%). The cases reached were 6,689 overall, whereas the chosen classes for the survey, two per institute, were second and fourth-year students.

Starting with the complete list of contacts, arranged by institute and territorial context, all school Directors were sent, well-before the official launch of the survey, an email inviting them to participate in the research initiative, comprising–other than the questionnaire file, which was subsequently digitalized for the purpose of online data collection–detailed information about the sponsoring bodies, and institutional entities formally involved in the project (University, Department, Observatory, scientific Coordination), about the scientific objectives, the research tools (techniques, tools, data analysis methods), and the intended use of the findings. A complex, careful reminder plan was provided for (three in all, sent out every 2 weeks of survey); furthermore, with the individual confirmation obtained, scientific collaboration effectively took shape on the basis of the active role of a teacher-representative for each relevant institute, an invaluable link between the research team and the interviewed students.

The survey took place in class during an entire period, wherein students from the selected classes (chosen by the school, on the back of the research group's indications, intended to reach a sample as ample and heterogenous as possible–in terms of variables like gender, nationality, academic performance etc.) completed the questionnaire online, mostly using their smartphones (in residual cases, with PCs or tablets provided by the school).

Recourse to the web survey, essentially “mandatory” in contingencies heavily conditioned by the restrictions dictated by the medical emergency, presented advantages and drawbacks. Typical web survey limits, including *sample mortality* and its *lacking statistical representativeness* (the sample reached is “self-extracted”), affect the present research occasion as well. However, as for the former aspect, it must be underlined that the element of “closure”, i.e., the inclusion of a *special population*–in this case Italian upper-secondary school students–constitutes (and has in fact constituted) an incentive to participation in the research. Moreover, the advantages connected with the use of such technique also fully characterize the present study: ample *sample coverage*; high *response rate*; *possibility of comparing*, subsequently, *the reached sample and the reference population* as to known characteristics; *curbing the cost of data collection* and *data entry*; *possibility of prompting sample subjects to respond*; *lower risk of social desirability of answers* and *higher drive for honesty* in case of intrusive questions (probably, in this latter regard, interviewees felt more comfortable in their assessment of the DL experience, as of their teachers' performance, in the absence of an interviewer).

The survey questionnaire is semi-structured and its web form was obtained through the platform *LimeSurvey*; it comprises 68 questions–including: closed, semi-closed and open questions; single-answer, multiple choice and battery–and presents as a fairly complex and intricate tool (numerous thematic dimensions were studied, and it contains several filter-questions).

As much as the sample reached cannot be considered statistically representative (the choice of second and fourth-year classes was not fortuitous), it is still notable that, when comparing the population and sample as per the variables *course of study* and *geographical area*, no particular unbalances emerged:

1. *Course of study in the population*– Gymnasium (High School Curriculum): 44.2%; Technical: 22.8%; Vocational: 21.3%; Mixed Institutes: 11.7%; *Course of study in the sample*–Gymnasium (High School Curriculum): 42%; Technical: 27.8%; Vocational: 25.4%; Mixed Institutes: 4.8%;
2. *Geographical area in the population* – Northeast: 14.9%; Northwest: 28%; Midland: 24.4; South: 20.8%; Islands: 11.9%; *Geographical area in the sample*–Northeast: 15.3%; Northwest: 23%; Midland: 25.8; South: 28.2%; Islands: 7.7%.

As mentioned, the study touched upon different aspects of young people's daily lives (from their school curriculum to family life, from uses of spare time to utilization of digital platforms, etc.), including the DL experience; the object of the work is to evaluate the *impact of DL on the students' lives*, actualized in terms of the perception of said expression of training, in a comparison of DL and in-person didactics as per three main spheres: *sociality* (interaction and relations with peers and educators), *energy invested* (time management, school commitment, study load), *efficacy* (appeal, acquisition of knowledge and competencies), *performance*.

Among the agents of influence considered to evaluate the weight of DL on studies, are:

1. *Socio-demographic variables* (gender, age, region/area of residence, nationality, composition of family unit, family's cultural capital, parents' job, etc.);

2. *Environment in which DL takes place* (available technology–in the household or school–and characteristics of the living space, oftentimes shared, throughout the COVID-19 emergency, with other subjects in DL and/or Remote Working frameworks).

3. *Familial atmosphere* and *system of relations/social and cultural opportunities fostered in the household*;

4. *School performance* and *quality of relationships in the context of school*;

5. *Usage of digital platforms, social networks and gaming*.

Finally, it appears gainful and appropriate to point out that within the questionnaire, are few and specific questions relating to DL, as there are few, targeted queries regarding the availability, at home and on the respondents' school campus, of adequate equipment (internet connection, e-learning platforms and digital devices in use), as a starting point for dealing with the emergency from a learning perspective. For obvious reasons of instrument economy (filled, furthermore, with references to multiple area of young people's daily lives), the questionnaire does not aim to retrace prior instances of the implementation of the digital in Italy's upper-secondary schools (in terms of investments predating the pandemic toward digital innovation and the acquisition of digital culture at school), nor the didactic methodologies adopted in the participants' study contexts. In the face of said limits, the questionnaire attributes the utmost relevance to the interviewees' assessments of the experience; moreover, a rich selection of questions aims at accounting for the interviewees' social profiles (with a particular view to: socio-cultural capital; material living conditions; affective, cognitive and relational resources of the subjects reached), a background that is variable and, simultaneously, essential to carry the load of an unprecedented emergency.

## Results

### Interviewees' opinions and assessments 1 year later: From the items to the factorial axes

A year from the outbreak of the Coronavirus emergency, the participants were asked to state their *preferred mode of didactic issuance*: within a picture that appears decidedly complex, the highest percentage of responses indicates a predilection for traditional in-person didactics (45.6%); followed by a preference for mixed didactics (30.6%), then the favoring of distance learning (23.8%).

**Figure 1:**
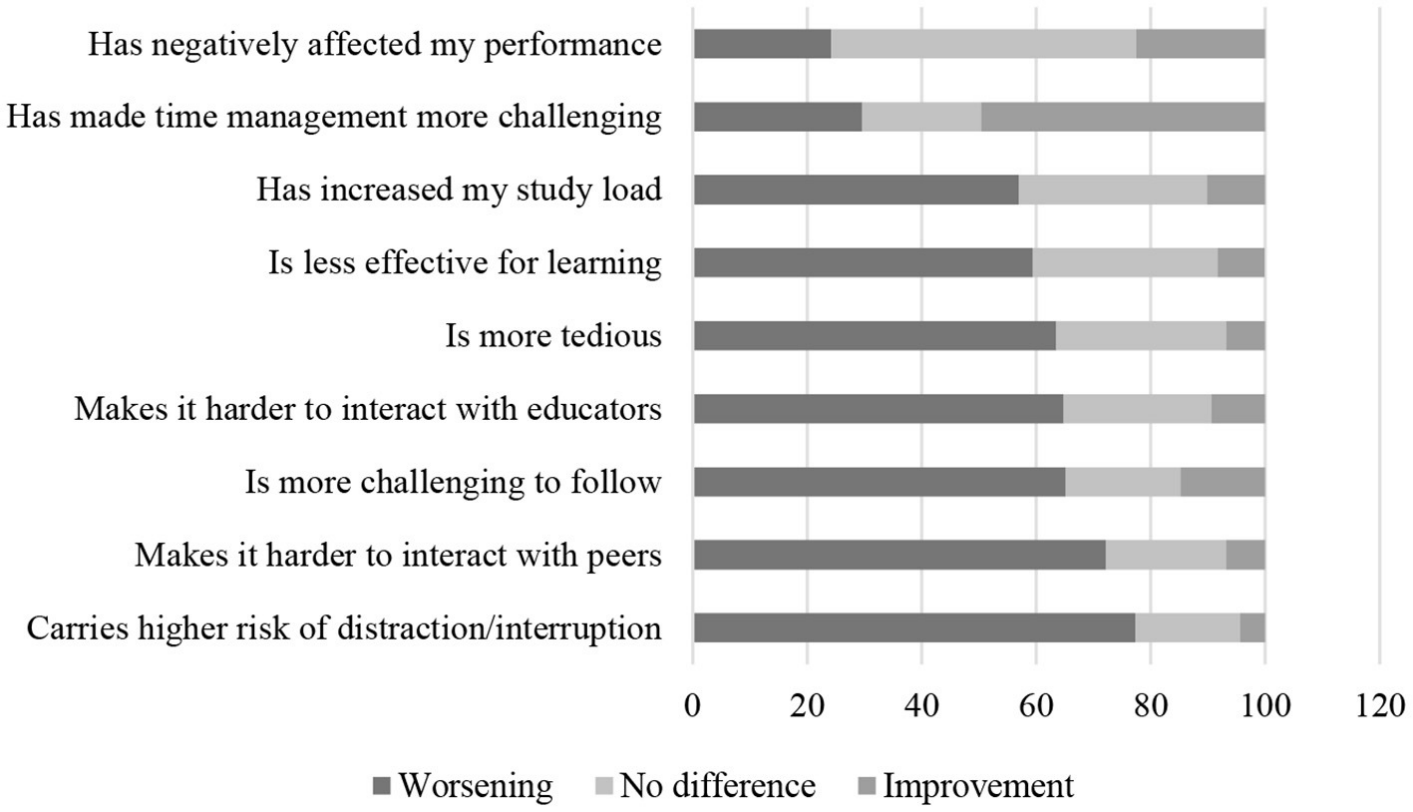
Opinions on DL.

Opinions on DL–which represent assessments bearing a specific reference to personal experience, not generalized judgments on the effects of DL on the figure of the student and school as an institution–are analytically depicted in the ribbon chart below (Figure 1). As can be seen, DL increases the risk of distraction and disruption, and negatively and transversally affects interactions between peers and with educators, leading to a decline, in one in four cases, of academic performance. However, the latter remains unchanged in 53.3% of cases–the highest percentage of consistency in the comparison between the pre-pandemic and pandemic phases–and improves 22.5% of the time. In terms of the positive effects of DL, noteworthy is the substantial percentage of responses (49.6%) concentrated on the option “allows for better time-management.”

The application of *Multiple Correspondence Analysis*, based on the 9 items referring to the assessment of the DL experience, allowed for the creation of an interesting synthesis of the pool of starting variables. Two factors were identified, which respectively explain 35.26 and 13.05% of the common inertia, the denomination for which was derived from careful overall reading of coefficients and factorial coordinates:

1. *Impact on the efficacy of the course of study* (appeal, learning and acquiring competencies) *and on the quality of teacher/student relations* (negative pole: no/positive pole: yes);
2. *Impact on the study load, performance* (timely progression, marks received) *and on the quality of peer relations* (negative pole: yes, increase and declining performance, but no difficulty interacting with peers; positive pole: no increase nor declining performance, but difficulty in interacting with peers).

### DL adaptation profiles: The influence of the physical and socio-cultural environment

Based on the extracted factors, through the application of *Cluster Analysis*, four main DL adaptation profiles were identified. Observing the internal composition of the groups that emerged, an attempt was made to *gauge the actual weight* of DL, *beyond the estimated, perceived, not always fully conscious one* supplied by the recorded answers. With the following tables (Tables 1–**4**), where the *test-values* are listed in descending order of importance (more specifically, they show coefficients higher than 2 in absolute value, i.e., those highlighting statistically significant associations between groups and active and illustrative variables-modalities), the process of interpretation and labeling of the emerged groups can be retraced (see the key below Tables 1–4 for the distinction of critical aspects, and not between active and illustrative variables-modalities). The review of the individual groups that emerged, yielded the following findings:

- *First Group*: *Adversarials* (24.9%–Table 1)–These subjects present serious distress both on the educational and relational levels, which hinders any and all future projects, and seems to imply the compounding of past and present hardships. Their *attitude* toward DL is one of complete *rejection* (hoping for a return to in-person didactics). These are socially vulnerable subjects, more often from traditional High School curricula, which bear the brunt of serious deficits and problems both at home and at school. A return to school is therefore perceived as the only deterrent in the face of difficulties that appear increasingly unsurmountable.
- *Second group*: *Dialectics* (36.9%–Table 2)–These are students who suffer most of all on a relational level (it is a problem of the present pandemic times, not a legacy of the past), who miss face to face interaction with their peers and their educators. They present a strong penchant for studying, and their *dialectic* approach is *of an adaptive type* (their predilection is for in-person didactics, but they are open to mixed didactics). Theirs is evidently not an open conflict with DL, seeing their capacity to keep their interest and motivation for study alive, to complete a successful course of study and design their future in higher education. These students, geographically located in Italy's Northeast (equalized, plausibly, by a local society which fruitfully invests in education and scholastic organization), are the strongest high school students, socially (employed parents, elevated cultural capital etc.), academically (consider the data on academic performance, prior to and throughout the pandemic) and emotionally (solid family bonds, quality relations at school etc.). The critical approach of the *Dialectics*, ≪*deprived of quality relationships*≫ and brilliant academic records, implies an awareness of the consequences, and not just in the short term, of DL; in the face of a strong connection to school (as to values, content, actors), they, in practice, resent the form didactics took on during the emergency phase.
- *Third Group*: *Consensual Critics* (18.7%–Table 3)–This group of students, despite favoring DL (and not excluding mixed didactics), seem to be having trouble due to increased work load, the greater effort required for educational activities, time management; in view of their prior instrumental/Heterodirected choice of their course of studies they are burdened, all things considered, by the weight of DL, with no support from their family of origin.

**Table 1:** First group (24.9%): Adversarials.

**Active/illustrative variable label**	**Significantly associated modality**	**Test-value**
DL makes time management more challenging	Yes	47.81
DL increases the study load	Yes	36.70
DL leads to a decline in performance	Yes	36.23
DL requires more effort	Yes	35.39
DL undermines the learning process	Yes	35.31
DL is more tedious	Yes	33.50
DL makes it harder to interact with educators	Yes	30.51
DL carries greater risk of distraction	Yes	24.78
*Preferred didactics*	*In-person*	24.69
DL makes it harder to interact with peers	Yes	15.50
*Changes in own social life on account of the pandemic*	*Used to be satisfying, now unsatisfying*	9.70
*Assessment of suitability of available space in the home*	*Unsuitable*	9.55
*Degree of confidence in teachers*	*Low/None*	7.33
*Relational criticalities at school*	*Problems with peers/teachers*	6.97
*Gender*	*Female*	5.92
*Quality of familial atmosphere*	*Ambivalent/Negative*	5.04
*Satisfaction as to free time in the family modality*	*Low/None*	4.89
*Number of electronic devices in the household*	*At least one*	4.63
*Able to rely on parents and teachers if necessary*	*Somewhat on teachers*	4.04
*Comparison of the modality free time in with family/others*	*Fewer opportunities for leisure*	3.92
*Satisfaction as to free time in the family modality*	*Satisfied to some degree*	3.50
*Composition of family unit*	*Absent parents – one parent present/yes siblings*	3.12
*Type of institution*	*Gymnasium (traditional HS syllabus)*	2.89
*Number of people that can be relied on if necessary*	*Small*	2.50

Critical aspects active variables

No critical aspects active variables.

Illustrative neutral/positive variables

*Illustrative variables negative connotation*.

**Table 2:** Second group (36.9%): Dialectics.

**Active/illustrative variable label**	**Significantly associated modality**	**Test-value**
DL makes it harder to interact with peers	Yes	32.38
DL makes time management more challenging	No	31.05
DL increases the study load	No	30.16
DL carries greater risk of distraction	Yes	29.74
DL makes it harder to interact with educators	Yes	29.68
DL is more tedious	Yes	27.80
DL undermines the learning process	Yes	27.33
DL leads to a decline in performance	No	14.89
*Preferred didactics*	*In-person*	8.81
*Preferred didactics*	*Mixed teaching*	5.15
Distance Learning requires more effort	Yes	3.26
*Post-graduation prospects*	*University*	6.09
*Academic record*	*Good/Excellent*	6.05
*Type of institution*	*Gymnasium (traditional HS syllabus)*	6.04
*Parents' employment* *situation*	*Employed*	5.38
*Quality of familial atmosphere*	*Positive*	5.35
*Assessment of suitability of available space in the home*	*Suitable*	4.96
*Relational criticalities at school*	*No issues*	4.95
*Geographical location*	*Northeast*	4.67
*Degree of confidence in teachers*	*High*	4.58
*Nationality*	*Italian*	4.11
*Geographical location*	*Midland*	3.33
*Cultural capital of family*	*High*	3.29
*Composition of family unit*	*Both parents present/yes siblings*	2.99
*Intensity of family life in social and cultural terms*	*Medium-high*	2.95
*Individuals in Distance Learning or Remote Working frameworks present in household*	*Presence of RW*	2.94
*Satisfaction as to free time spent in the family modality*	*High*	2.76
*Choice of secondary school*	*Self-directed*	2.75

Critical aspects active variables.

No critical aspects active variables.

Illustrative neutral/positive variables.

*Illustrative variables negative connotation*.

**Table 3:** Third group (18.7%): Consensual critics.

**Active/illustrative variable label**	**Significantly associated modality**	**Test-value**
DL increases the study load	Yes	23.91
DL undermines the learning process	No	23.27
DL makes it harder to interact with educators	No	18.63
DL is more tedious	No	18.22
DL makes it harder to interact with peers	No	18.13
*Preferred didactics*	*Distance Learning*	**10.70**
DL carries greater risk of distraction	No	7.82
*Geographical location*	*South*	4.88
DL makes time management more challenging	Yes	4.82
DL leads to a decline in performance	No	4.41
*Preferred didactics*	*Mixed teaching*	**3.71**
DL requires more effort	Yes	3.56
*Nationality*	*Not Italian*	3.69
*Composition of family unit*	*Absent parents – one parent present/no siblings*	2.69
*Quality of familial atmosphere*	*Ambivalent/Negative*	2.63
*Choice of secondary school*	*Heterodirected*	2.47

Critical aspects active variables.

No critical aspects active variables.

Illustrative neutral/positive variables.

*Illustrative variables negative connotation*.

Observing, in synthesis, profiles 1 and 3, these seem to receive the full force of the negative impact of DL, and the *interpretative key of prior deficits, material and relational*, may apply. It in fact appears that the students most afflicted by DL are those:

- With troubled academic records;
- Non-Italian;
- Whose families experience serious economic and employment issues;
- With prior problems with relations, family and school;
- Struggling in terms of available household spaces (for studying and personal leisure) and with limited opportunities for entertainment and cultural growth.

DL, therefore, entails *the scaling of future projects on part of the* very *subjects who are* ≪*already*≫ *vulnerable*, who are at higher risk of dropping out of school, and of social exclusion.

*Fourth Group*: *Consensual Opportunists* (19.5%–Table 4)–These subjects present an *adaptation to DL, on the educational and relational fronts*, based on an *instrumental-acritical type of consent*. In the eyes of these students, typically from the South of Italy (like *Consensual Critics*), DL appears as an efficient solution, with a view to quickly wrapping up, without too much effort, their time in school and break speedily into the world of work. In terms of these participants–*their audio and video* presumably unfailingly *turned off*, whose attendance during lessons and, more generally, at school is anything but active – the impression is that an individual acritical approach, and an opportunistic one lacking farsightedness have insidiously combined, possibly with *the help of the faculty* (perhaps not overly concerned with monitoring the attention and participation of the student body). The collected answers point toward a decrease in study load, a surprise indeed in contexts such as technical institutes, known for exposing students to workshop, like technical-practical, activities.

**Table 4:** Fourth group (19.5%): Consensual opportunists.

**Active/illustrative variable label**	**Significantly associated modality**	**Test-value**
DL is more tedious	No	48.60
DL undermines the learning process	No	46.98
DL makes it harder to interact with educators	No	46.65
DL carries greater risk of distraction	No	45.48
DL requires more effort	No	41.31
DL makes it harder to interact with peers	No	32.43
*Preferred didactics*	*Distance Learning*	28.58
DL makes time management more challenging	No	26.04
DL increases the study load	No	22.87
DL leads to a decline in performance	No	22.51
*Changes in own social life on account of the pandemic*	*Satisfying in past and present*	8.63
*Geographical location*	*South*	7.96
*Type of institution*	*Technical institute*	6.60
*Post-graduation prospects*	*Employment*	6.51
*Gender*	*Male*	6.49
*Assessment of suitability of available space in the home*	*Suitable*	5.22
*Parents' employment situation*	*Unemployed*	3.92
*Academic record*	*Satisfactory*	3.85
*Type of institution*	*Vocational*	3.77
*Choice of secondary school*	*Heterodirected*	3.68
*Relational criticalities at school*	*No issues*	3.37
*Satisfaction as to free time in the family modality*	*Satisfied*	3.33
*Parents' employment situation*	*One parent unemployed*	2.95
*Intensity of family life in social and cultural terms*	*Low*	2.55

Critical aspects active variables.

No critical aspects active variables.

Illustrative neutral/positive variables.

*Illustrative variables negative connotation*.

These kids, evidently projected toward the professional world, are eager to find a job and, simultaneously, come from family contexts that struggle in this regard. In view of a behavior that appears to be institutionally tolerated at higher levels as well, they experience DL as a *cushy parking area* in a manner that's not problematic or conflictual. However, they are also at risk of exclusion: *DL as an expedient* and *exit strategy* vis-a-vis school, perceived as a hindrance, which also reflects a strong connection between socio-cultural and geographical contexts and schooling (Bourdieu, 1984), could end up undermining the traditional functions of school and the role of teachers, as well as condemning those kids trapped in this spiral to *likely social descent*. Scant commitment and studying, the degradation of the content and activities provided, school marks perhaps stemming more from ritualistic practices than actual assessments of performance, cannot but result in modest academic profiles devoid of any specific skills, and not too enticing on the job market.

### DL adaptation profiles: Influence of the digital environment

The available data paint a fairly eloquent picture in terms of the interviewees' usage of technology. Before reviewing the findings regarding the usage of digital platforms and social networks for non-educational purposes, it is certainly worth dwelling on a critical aspect directly connected to the DL experience, the foundation, as it were, of a process of adaptation to it, or lack thereof.

Starting with *Internet connection*, essential to access online lessons and to complete assigned homework and tasks: in reference to their own *household*, it is viewed as inadequate by 14.2% of participants, in reference to *the school campus*, it is defined as lacking by 61.6% of them (a twofold negative review, at home and at school, is found in 8.7% of cases); these are clearly remarkable percentages if one thinks the survey did not take place at the start of the pandemic, but in its second year [see the 2021 Youth Report (Rapporto Giovani), published by the Toniolo Institute]. Either way, the perceptive data presented herein on the dimension of the *density/functionality of the connection* would need to be compared to their objective counterpart, encompassing a targeted assessment of the current technological equipment in Italian schools. Less problematic are the data regarding the available technology in the household: among the available devices laptop computers stand out (85.6%), followed by tablets (57.4%) and desktop computers (38.9%); only 2.5% of participants appear to have no devices (excluding smartphones, which were available to all the contacted interviewees). *Adversarials* are the most affected students in this regard as they, above others, lament precarious internet connections both at school (35.9% vs. values between 17.1 and 28.4% for the other profiles) and at home (32.6 vs. 17.3–18.1%). The *problem of “access” to the internet and to technology*, along with that of *inadequate household spaces for studying-leisure*, when present, *exacerbate all other identified issues*. In contrast to the school's subpar performance, most households were instead found to have *electronic devices* (one in 45.4% of cases; more than one for 35.2% of them) and *an internet connection*.

Moving on to the hardware and software components, and focusing on the digital environment in which the young participants are located, the traits of *diffusion* (or better still, *immersion*) and *transversality* with respect to *access and usage to/of entertainment platforms for streaming movies, TV shows and music* appear glaring within our sample: 95.2% of respondents state they access at least one of the listed platforms (Netflix, Amazon Prime Video, Disney+, Sky, Spotify, Infinity, TIMvision, Dazn, YouTube Premium, Now TV, Discovery+ e Apple Music) and nearly one third (30.1%) say their family has five subscriptions or more. Accessing audio-video content, more than a leisure activity of a personal nature, is above all an opportunity to share and compare in identity and relational terms (Caneva, 2012; Coviello and Re, 2020), capable, by virtue of the web and social platforms, of feeding and consolidating networks of interactions and connections, and specific skills likewise, both within and without the family context.

The interviewees were also asked to rate, by importance, the most utilized social networks and apps. Whatsapp comes out on top, followed by Instagram (the use of both Whatsapp and Instagram pertained to about 90% of interviewees, while 60% or so of the sample was characterized by the combined usage of said apps). TikTok and YouTube follow, then, residually, the remaining platforms. By aggregating all the data, *instant messaging platforms* amount to 33.6% of all answers and *social networks centered around images* (among which Instagram prevails) embrace 32.4% of the collected responses. In third place are *social networks for videos and live streams* and, residually, *platforms for online meetings and non-descript social networks*.

Where all the teenagers in the sample use social platforms in general, nearly all of them have an Instagram profile. It in fact surfaced that 1 student in 10 does not use Instagram, around 20% of respondents are characterized by reduced usage of said platform (up to 1 h - we here find 40% of *Dialectics*, in the face of percentages between 15.4 and 28.4 in other groups), just over 40% by moderate usage (1–2 h), the remaining 30% or so by substantial use (2 or more h per day). The latter group comprises the sub-group of the hyper-connected, who have claimed to spend over 5 h on Instagram each day, right up to estimates in the double digits.

In line with national and international studies on young people's use of Instagram, the presented case also saw the widespread and transversal use of the platform, its assiduous, sometimes pervasive consumption, a certain pull toward acquiring the status of influencer, a tendency for *showcasing* as an instrument to construct and reinforce the ever-growing social and media exposure (Codeluppi, 2021). Findings, reported in detail below, which highlight the need for an increasingly active and prepared involvement in the lives of growing youngsters–for whom digital technology and internet connection are the norm–on part of families and the world of education. Girls more than boys (31.1 vs. 25.5%), like students in vocational institutes (36.4%) more so than those in traditional Gymnasium-type curricula (25.1%) and technical schools (29.5%)–who are clearly subjected to a heavier study load–are characterized by a particularly substantial use of Instagram daily. An occasionally excessive consumption of this app (which, it must be recalled, is used in combination with other platforms) is also associated with unsatisfactory school performance (37% of cases, compared to 31.9% of subjects with satisfactory achievements and the 24.1% who average good or excellent marks).

The participants were asked, moreover, to list the number of their Instagram followers and following, with the purpose of assessing the breadth of their network on said platform, and grasp the diffusion of *potential influencer* profiles, subjects, in other words, capable of conditioning their reference community, evidently by virtue of their power of communication and aggregation, and engaged in building their reputation within their reference network. The resulting picture shows the interviewees clearly immersed in copious networks, and a good number of them inserted into the category of potential influencers; it shall suffice to consider that around 15% of students can claim over 1,000 following, and ~20% presents over 1,000 followers.

**Figure 2:**
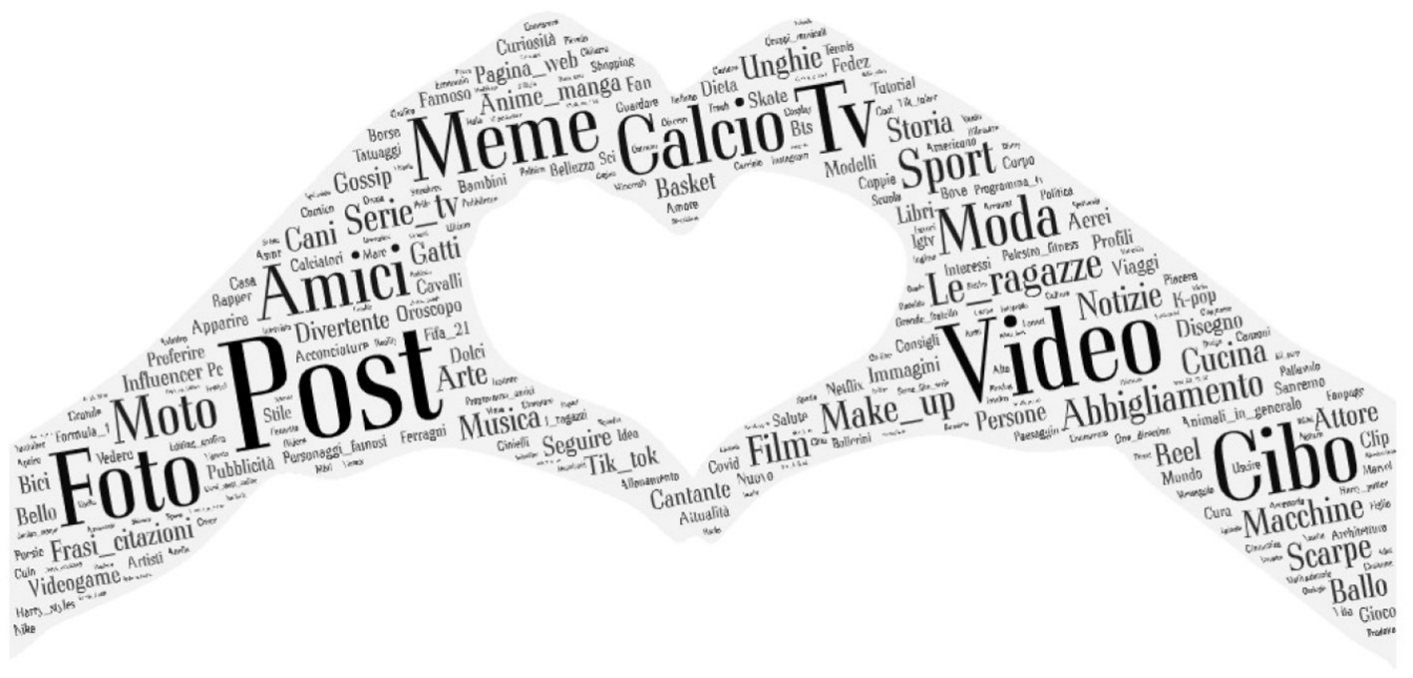
The instagram “browse tab”: keyword cloud.

The questionnaire comprised an interesting question (*Open your Instagram “browse” tab now, what are the first three things you see?*), aimed at grasping what, amidst the contacted students, the main uses of the platform were. On the open-answer format of the 5,536 valid cases totaled (of the 6,689 contacted through the survey), statistical-textual analyses were carried out. The text comprised 39,240 occurrences, including 3,717 distinct forms. By using a mixed procedure, partly automated and partly customized, 385 keywords were identified. These, essential to a correct breakdown of the functions attributable to Instagram, are presented in the following word cloud (Figure 2).

Besides generic words like post, video, meme, photo (which each recur between 900 and 1,000 times), it is notable how the pillars of young people's searches on Instagram orbit, essentially, the following focal points: soccer, clothing, friends, food, girls (400 occurrences and above); fashion, make-up, sports, TV shows (300–399); motorbikes, anime-manga, quotes and phrases, shoes, news, nails (between 200 and 299).

The analysis of the data available on Instagram ends with a reference to the linguistic specificities surfaced in the analysis of the textual corpus as per the DL adaptation profiles: 1. *Dialectics* present a use of the platform that is especially complex, varied and sophisticated, in which the mainstream media and real life find ample room–their searches comprise several *sporting disciplines, music festivals, art, current events*; 2. *Adversarials* feature persistent references to *fashion* and well-known *brands, make-up, star signs*; 3. *cartoons, gaming, drugs, engines* prevail in the C*onsensual Critics'* searches; 4. similarly, *gaming, soccer, MotoGP, celebrities* are the specifics of the *Consensual Opportunists'* profile.

Moving on to the data collected on the dimension of gaming, it can be said that a large chunk of *non-*players (38.2%) counterbalances an even greater number of *gamers*, assiduous and passionate to varying degrees (the remaining 61.8%). The *occasional gamer* profile recurs in nearly 30% of cases, followed closely by that of the *assiduous gamer*, who dedicates at least 1 h a day to this dimension, up to a maximum of 4 h (25.1%). At the bottom, *hardcore gamers*, who allocate a substantial number of hours to videogames each day (at least 4, but in some cases 8 or more).

Amidst the *subjects removed from the gaming world* there is an abundance of females (62.9 vs. 11.6% of males), high school students and those enrolled in vocational schools (45.4 and 42.7 vs. 26.2% for technical institutes), students with a good or excellent academic record (44.3% vs. percentages between 28.2 and 33.2 for profiles with lower marks), those subjects intending to continue their studies and oriented toward higher education (45.6 vs. 27.9%–subjects intending to seek employment after graduation–and 30.9%–subjects who are undecided or intending to take a break after completing secondary school), interviewees with a particularly intense social and cultural family life (in the dichotomous capacity of the index: 42.2 vs. 33.6%).

Among the assiduous and hardcore gamers boys prevail over girls (respectively 43.7 vs. 7.7% and 12.9 vs. 2.5%), technical institute pupils (33% vs percentages between 20.4 and 21.8 for assiduous gaming, and 11.2% vs values between 4.5 and 8.3% for the hardcore tier) and *Consensual Opportunists* (32% of hardcore gamers vs. values under 23% for other clusters); moreover, it would seem that extreme or assiduous gaming has a negative impact on academic performance, while being contemporaneously connected with the prospective of leaving education after secondary school, and with a more modest cultural input on part of the family of origin (*p* = *0.000*). It should be noted that the degree of satisfaction relating to the quality of free time spent within the family is fairly high/very high in 80% of cases, in an almost transversal manner. In the case of *Consensual Opportunists*–hypothetical DL users with no audio or video, but also passionate and frequent multi-platform users–the feeling is that the faculty's tolerance of certain academic behaviors, is met with just as much carelessness, if not general laxity in the household, a source of satisfaction for these youngsters. The abovementioned figures lead to the hypothesis that an opportunistic conduct may be more widespread than what was actually recorded. What emerged seems to lend itself to be interpreted as a form of *gratification connected with meeting particularistic and “surface” needs, disconnected from the achievement of concrete, personal educational-growth objectives*. In the face of these facts, the doubt creeps up that the *household environment* may be so broadly permissive, that it could be defined as *tailored to the youngsters' needs*. There is no shortage of particularly conscientious and responsible youngsters; however, most probably take advantage of such freedom, which in the long term is detrimental, as well as very risky.

The information at our disposal ends with references to preferred gaming mates. One fourth of interviewees preferred *playing solo*, the majority (54%) claim they *share gaming with real-life friends*. The remaining participants, in similar proportions, which in both cases are just over 10%, prefer on the one hand, games they share with their family members (real-life subjects, siblings in particular), on the other, gaming practiced within the reference *virtual community* (the latter surfaces as a typical trait of *Consensual Critics*-*p*=*-000*). While assiduous gamers tend to select real-life friends for online games, thus fully hybridizing the two spheres of life, *hardcore* gamers, conversely, tend to prefer relations and *gaming* experiences that originate within the Web, and develop and take form therein (25.8% vs. percentages between 5.9 and 11.2% for other interviewee profiles).

The available empirical base encourages the problematization, and critical observation of extreme exposure to the world of *gaming*, which–within a process where it is a causally relevant factor (for example, with respect to bumpy academic performance), or a factor with significant effects (as a fallback for or reaction to familial and social contexts offering little stimulation or appeal to youngsters, resulting, in extreme cases, in forms of social withdrawal; the expression of a personal immersion into a given community; or still, a reflection of an existence heavily conditioned by the pandemic emergency)–can turn out to be an insidious source of depletion of resources (attention, focus, psycho-physical balance, time, money) and future prospective. By contrast, there are tangible elements to define most of the interviewees' approach to the world of *gaming* as responsible; in this sense, it could be interpreted as a cultural and cognitive resource, as well as a basis to form expectations vis-a-vis school, and for reviewing the performance of teachers/the degree of innovation of the instruments available in schools.

## Discussion

The empirical evidences presented herein lend themselves to reflection which, it could be said, enriches and elaborates the reading, confirmed^6^ on several fronts, of the DL experience centered on the theme of *digital divide*. As known, the pandemic emergency has deeply affected schools' priorities, suddenly moving the needs connected with, precisely, digitalization to the top of the list (it shall suffice to consider the government's substantial economic investments in 2020 and 2021^7^, aimed at improving the connectivity of educational institutes), albeit the results of such assistance did not always appear fully conclusive, nor evenly distributed on the territory. Moreover, with respect to the issue of digital competencies, closely connected with teachers, who represent the fundamental link for the transmission of educational content (and less connected, assuredly, with students, that is to say, the demographic of digital natives), it was found that half of Italian educators–who, furthermore, widely consider themselves poorly equipped in terms of using digital technology–were never exposed to practical and specialized forms of training devoted to innovative didactics of a *digital* stamp (OECD, 2020).

In the face of this, one can imagine the unease experienced by a large number of Italian educators, suddenly catapulted into the realm of distance learning platforms, and forced to direct old knowledge into new and unfamiliar, or wholly uncharted, channels.

Although the combined action of the abovementioned deficit factors, also considered in view of appropriate socio-territorial variables, could sufficiently explain most of the above findings, it is necessary to carry the ongoing reflection forward, transforming the surfaced evidence as to “the school of the pandemic” into a broader “lesson for the future” for the national education system. This, above all, based on the concerns expressed by the vast majority of students–including those who maintained brilliant academic careers throughout the pandemic, the motivated ones, inserted in scholastic/household contexts with effective technological equipment–in the condition of potentially optimal usage of DL.

As known, a wide range of webinar and videoconferencing apps (including Google Meet, Zoom, Cisco, Webex etc.), along with integrated system for module-based learning (such as Microsoft Teams and GSuite for Education), and instant messaging services (like Whatsapp, Telegram), comprise the most widely used “technology pack” for the issuance of DL (see Mascheroni et al., 2021). Though not excluding that said tools represent a fruitful channel for the transmittal of some content, which can also provide for the tangible learning results, it makes sense to question whether, and to what degree, platforms with said communicative-interrelation architecture can effectively reproduce complex experience compositions like *being at school* and *going to school*. Whereas, in fact, DL has also complicated and impeached the indispensable function of evaluating learning, the school dimension that in all “remote” contexts has suffered the gravest depletion is indubitably the social one, attributable to a system of peer, and teacher-student, relationships. Even when implemented in optimal conditions, as to connectivity and to educators' digital competencies, DL has generated an objective break between two processes which are inextricably connected in conditions of normalcy (acquiring academic-curricular knowledge and acquiring socio-relational competencies, needed in school and in life, in the present and future). Worse still it has, in fact, compromised the actualization of socio-relational dynamics, essential for solid, long-lasting transference of curricular knowledge as well (the majority of interviewed students found serious difficulty in communicating with their classmates and teachers throughout the pandemic).

Starting with this grave weakness, reflection on alternatives to DL becomes essential, projecting oneself in a future scholastic dimension that can safeguard, above all, the *human and relational components*, while still being receptive to technological advances. In such a scenario, one could think in terms of a profitable combination of school with virtual reality (VR), albeit the road ahead is certainly lengthy, considering the fact that DL was, in the Italian context–and at best–a mere transmittal of traditional lessons into a decidedly poor virtual environment, generally comparable to forms of individual MOOC-style training (see Head, 2014).

The impression is that in the last few years, notwithstanding a context of generalized investment toward digitalization, schooling has mostly ignored the powerful cues stemming from digital and technological innovation, remaining essentially anchored to the “classic”–or traditional, as some may prefer to say–models and systems of didactic issuance. The potential clash between innovation and tradition had not happened, remaining, furthermore, in a latent state of sorts on account of the surefire adaptation capabilities of the training's end-users, unfailing activators of a *switch off and on* procedure: students in class, digital natives on the outside. The pandemic broke that spell, producing an unseen short-circuit, the effects of which we have tried to report. Traditional education has had to, concretely and dramatically, endure the challenge of innovation and, for the first time, found itself having to enter and move exclusively on digital terrain, registering–as was to be expected–difficulties, delays, widespread and visible ineptitude, which the digital natives most assuredly noticed, they who, paradoxically, were perfectly up to the challenge. Many digital natives, for their part, found themselves, as seen, missing traditional models, having the new DL system caused many dysfunctions and a general depletion in terms of learning, as well as the essential socio-relational deprivation due to the disappearance of school classes, as physically intended. In this context, a return to normalcy–i.e., the reestablishment of the *switch off and on* model–for most stakeholders (institutes, students, families, policymakers) represented a moment of immeasurable relief. However, it would be very worrisome if the feeling of liberation from DL concealed even the slightest conception that digitalization and technological education are a hindrance to scholastic educational processes.

The potential intersection between the scholastic realm and VR would in fact imply a veritable paradigm shift, keeping in mind that VR, in the virtual reproduction of swatches of the real world, would allow its end users to move in an artificially created, computer-generated three-dimensional environment, and to interact with simulated objects and people (avatars) (Burdea and Coiffet, 2003; Steinicke, 2016; Kamińska et al., 2019). As known, access to VR is generally controlled *via* an HMD (Head-Mounted Display) device, with an integrated display and lenses, which confers the user with three-dimensional vision; having donned the HMD, the user is then able to experience, as if in an actual physical space, immersion into a digital environment, sometimes experiencing sensory stimuli so ample and profound (visual, auditory as well as touch, smell and taste-related), as to allow VR to be defined as the technology of the three Is (Immersion, Interaction, Imagination–see Burdea and Coiffet, 2003). Shifting focus onto the core aspect of our interest, the process of acquiring academic competencies, it is possible to state that the areas where VR could reach its maximum degree of expression are the following: engaging students; learning style (potentially, a particularly active and constructive one); empathic charge in the learning experience; exercising creativity and the capacity for abstraction (Hu-Au and Lee, 2017).

The hypothesis herein is an application of VR, as much to the classroom's physical space, as to the object-subject of the lesson, in an effort to reproduce the entire school classroom virtually (including student and teacher desks, boards, other teaching aids, etc.), wherein the avatars of the students and teachers in attendance act and interact as per styles and dynamics based on actual school life. Resorting to said technology, which is evidently capable of prompting substantial cognitive and emotional engagement, would, on the one hand, be finalized to limit, as much as possible, the household isolation of students which started with the pandemic emergency, and on the other, to contrast those practices which, as our data attest, have severely reduced the reach of DL (cameras off, parallel engagement in chatting, simultaneous execution of other activities, etc.). The promulgation of experimentation of VR technology in said direction is, from the authors' point of view, desirable albeit, as known, the operating costs of VR are still too high, and the potential effects of tolerance-building on the users (loss of motivational drive, physical illness, etc.–see Kavanagh et al., 2017), especially on the back of prolonged usage of HMDs, must not be overlooked. Furthermore, the VR technology sector is in very rapid expansion (to this effect, a new version of HMD glasses–more wearable and comparable to said accessory's customary models–is among the short-term expectations), partly by virtue of the exceptional vitality of market leaders such as HTC, Valve, Oculus, Google, Sony. Facebook's 2014 acquisition of Oculus, an active startup in the production of VR technology and HMDs, and the founding, by Zuckerberg himself, in late 2021, of the multinational Meta Platforms Inc., are within this scenario of rapid technological advancement, and their mission concentrates around a few keystone principles^8^–interaction/co-penetration of the real and virtual realms; social connection; economic, social and environmental sustainability; balancing wellbeing, work and life needs; breaking down inequalities–which would seem to imply the transference of VR technology testing into broad–and not niche–segments of society (Beck, 1986; trans. it. 2000).

Clearly the possible introduction–efficient, of large proportions and shielded from the trap of technological virtuosity as an end in itself -, of VR technology into the field of education implies the adoption of a multidisciplinary collaboration between disciplines (such as IT, mathematics, sociology, educational psychology). On the other hand, one cannot gloss over the consequences, in ethical terms as well, of the testing encouraged herein, on the back of the survey results emerged (Falck et al., 2018). As previously mentioned, VR experiences imply a fairly elevated level of sensory, cognitive, emotional and physical engagement, especially in view of activities, such as DL, which are prolonged and systematic. Therefore, in the face of likely favorable initial responses, it is fundamentally important to be able to gauge the potential tolerance-building effects of such equipment, and all the more reason to monitor the impact on human health, in terms of the different physio-psycho-sensory disorders, of intense usage of VR technology and devices. Nevertheless, faced with a technological advancement that appears constant and inexorable (and which, regardless, will never completely annul the distance between real and virtual), it is reasonable to believe the scale of such disorders may gradually decrease, leaving room to the extensive, desirable testing of innovative didactic forms based on VR.

For these reasons, possible developments in the direction of VR seem to be, at least on paper, very promising, capable of pushing back against forms of skepticism with respect to digital schooling, partly fed by the unproductive DL ordeal. As the authors have tried to argue, VR is not just a strictly technical approach to the transmittal of knowledge that, for many school subjects, and not just of the strictly applied type, seems to have a competitive edge over more traditional teaching modalities. VR, above all–and this is the element of most interest in the authors' view–seems to provide a valid design base to the possibility of remotely recreating at least the semblance of a class-group, thus achieving the reduction of the damage done on the socio-economic front to which the videoconferencing system, that characterizes DL, inevitably leads.

Finally, VR could perhaps succeed in closing the gap and reducing potential conflicts between students and digital natives, that is, two sides of the same coin: the young users of institutionalized educational procedures. Just as DL was a missed opportunity of sorts, even resulting, where possible, in an increase of the student-digital native rift, except for a subsection of youths who–as seen–employed it in a chiefly opportunistic manner–VR could embody a veritable moment of vindication for the digital, as it were, as it would offer its undoubted service potential to users capable of its competent and efficient usage. A vindication that would deserve to be tested and actualized swiftly, without awaiting other–undesirable–epidemics or comparable quasi-apocalyptic occurrences that would bring the same isolation effects priorly endured, and which unfortunately cannot be excluded in view of a society which seems unprecedently exposed to such risks.

In conclusion, returning both to the premises of this work, and to the findings, extensive training on, and efficient monitoring of, the risks and benefits of new technologies requires constant *dialogue* and *discussion* between the relevant actors and agencies (starting with that *between generations*–at the moment, *parents-children* and *teachers-students* appear too distant), as well as *clear and common rules* on the phasing and manner of use of digital technology, on the content it applies to, and on the meetings and exchanges it shall beget. Forms of opportunism and short-sightedness associated with the young which the data have revealed, like the inferred expressions of tolerance, laxity and/or carelessness attributable to the adult world, bring the authors to highlight the importance of a more cognizant tutelage of youngsters on part of their parents, and of students on part of the educators. This takes concrete shape in constant and competent availability to *register and analyze expressions of unease and apathy*, implying a *dialogue on equal terms* and *rapport* between generations (starting with *competent and responsible practices* enforced by adults whom, at the moment, youths perceive as extraneous, in effect, to their world (see Livingstone, 2008; Boyd, 2011).

With a view to *laying the foundation for further agreements to be made*, looking beyond the pandemic, *the young, multitasking and hyper-connected*, as described in previous pages, need *care, closeness* and *empathic attention*, like they need *fresh motivational inputs*. Today, these appear to be novel challenges for the adult world, on the back of reasonable expectations on part of the youth. In terms of these legitimate anticipations, what will not suffice are attempts, illustrious as they may be, that are isolated and non-continual, as they evidently require systematic projects and investments, a common vision and intent, incentivized and supported in politics as well. Having bridged the gaps and the distance, and following a reasoned and productive investment in the area of competencies (including: schooling educators as to the platforms used by digital natives; inserting said platforms in the teaching method, etc.), these *newer forms of didactics* (which certainly do not coincide with current e-learning platforms), appropriately combined with in-person instruction, can go from being *hindrances* and *multipliers of difficulties* to representing *sweeping advantages* in addition to *efficient and transversal solutions*.

## Data availability statement

The raw data supporting the conclusions of this article will be made available by the authors, without undue reservation.

## Ethics statement

Ethical review and approval was not required for the study on human participants in accordance with the local legislation and institutional requirements. Written informed consent to participate in this study was provided by the participants' legal guardian/next of kin.

## Author contributions

MF was responsible for the sections entitled: introduction, materials and methods, and results–DL adaptation profiles: influence of the digital environment. AF for the sections entitled: results–interviewees' opinions and assessments 1 year later: from the items to the factorial axes; results–DL adaptations profiles: the influence of the physical and socio-cultural environment, and discussion. All authors contributed to the study design and approved the final version of the manuscript for submission.

## Footnotes

^1^These considerations are certainly valid, above all, for older educators, inevitably further removed, and not just chronologically, from the digital natives generation, and for faculty in primary and secondary schools, more than for university professors, who have a longer history (thus, before the advent of COVID-19) of involvement in the process of digitalization and innovation of didactics.

^2^On the innovative potential that recourse to digital holds in periods of “normalcy”, and with adequate degrees of design and planning, refer to the volume edited by Veletsianos (2016).

^3^There is another aspect that, at least, warrants a mention: the physical space where students utilized DL is found within the household, a space that is anything but neutral, inhabited by a varying number of individuals and, commonly, the setting for a variety of activities, not exempt from problematic aspects and forms of interference vis-a-vis the ordinary flow of the educational-training process.

^4^The study, entitled *Critical Thinking and Cognitive Populism in the Digital Platform Society*, is within the scope of activity of the Observatory of Electoral Sociology and the Department of Communication and Social Research (Scientific Director of the Observatory: Prof. Maria Paola Faggiano/Research Coordinator: Prof. Carmelo Lombardo).

^5^The complete updated list of the email addresses of upper-secondary Italian schools located in region and province capitals nationwide is available online on the website for the Ministry of Education (Source: *Open Data MIUR 2019-2020*).

^6^For further information on the official statistics relating to IT-digital equipment in Italian families, divided by the age of their offspring, by relevant territorial area, (the deficit is particularly problematic in the South), by the presence, or lack thereof, of differently abled students, etc., see Istat, 2020, 2021.

^7^The Italian government has deployed sizeable resources to support DL during the lockdown and the height of the pandemic; moreover, the PNRR (National Recovery and Resilience Plan) includes three transversal priorities shared by Europe at large, among which are *digitalization* and *innovation*, and comprises six essential missions, including the one denominated *Digitalization, Innovation, Competitiveness, Culture*.

^8^With specific reference to the realm of school, the same principles, and that of social inclusion first of all, long before the pandemic, were at the basis of Digital School Centers (CSD - established with bill n. 179 dated 18/10/2012), likewise, amidst the expressions of Indire (National institute for educational documentation innovation and research), the movement of Avanguardie educative (2014) and the Manifesto delle piccole scuole (2017), called to answer the needs of the student body on the islands and in inland mountain areas, at constant risk of social isolation and irregular in-person school attendance, on account of the environmental and weather conditions in relevant areas.
